# Sign of Leser-Trélat Associated with Esophageal Squamous Cell Cancer

**DOI:** 10.1155/2014/825929

**Published:** 2014-02-06

**Authors:** Vinaya Gaduputi, Chaitanya Chandrala, Hassan Tariq, Kalyan Kanneganti

**Affiliations:** Department of Medicine, Bronx Lebanon Hospital Center, 1650 Selwyn Avenue, Suite No. 10C, Bronx, NY 10457, USA

## Abstract

The sign of Leser-Trélat is a rare paraneoplastic phenomenon marked by accelerated onset of multiple seborrheic keratoses. The occurrence of the sign often points towards underlying visceral malignancies which in a majority are adenocarcinomas of the gastrointestinal tract. We report this case of a 65-year-old man who presented with sign of Leser-Trélat and was diagnosed with poorly differentiated squamous cell cancer of the esophagus. To our knowledge this is only the second such reported association of Leser-Trélat sign with squamous cell cancer of esophagus.

## 1. Introduction

The sign of Leser-Trélat is a misnomer as both Edmund Leser and Ulysse Trélat described skin lesions unrelated (senile angiomas) to seborrheic keratoses [1, 2]. It was Hollander that first recognized the possible association between worsening seborrheic keratoses and underlying visceral malignancies [1]. The sign of Leser-Trélat is a rare paraneoplastic phenomenon marked by accelerated onset of multiple seborrheic keratoses. This acceleration can manifest both as increase in size and number of the skin lesions. Validity of the sign is subject to contestation [2, 3] as both visceral malignancies and seborrheic keratoses increase in incidence in parallel, with advancing age. Case control studies could not demonstrate a strong association of seborrheic keratoses with underlying visceral malignancies [3–5]. However, it has been noted by Schwartz [6] that there is a strong association of sign of Leser-Trélat with malignant acanthosis nigricans, a well established paraneoplastic lesion, and therefore could itself be regarded as a paraneoplastic phenomenon. Multiple cases have been reported about rapidly growing seborrheic keratoses with various underlying malignancies, commonly including adenocarcinomas of stomach, colon, and lymphomas.

## 2. Case Report

**Figure 1:**
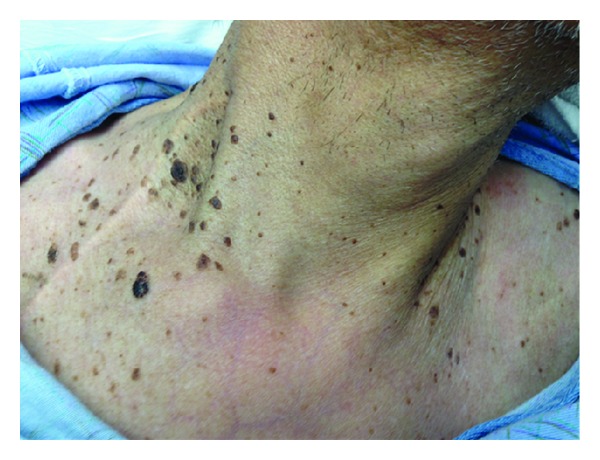
Multiple hyperpigmented, well-demarcated and raised lesions with “stuck-on” appearance are seen on both sides of the neck.

**Figure 2:**
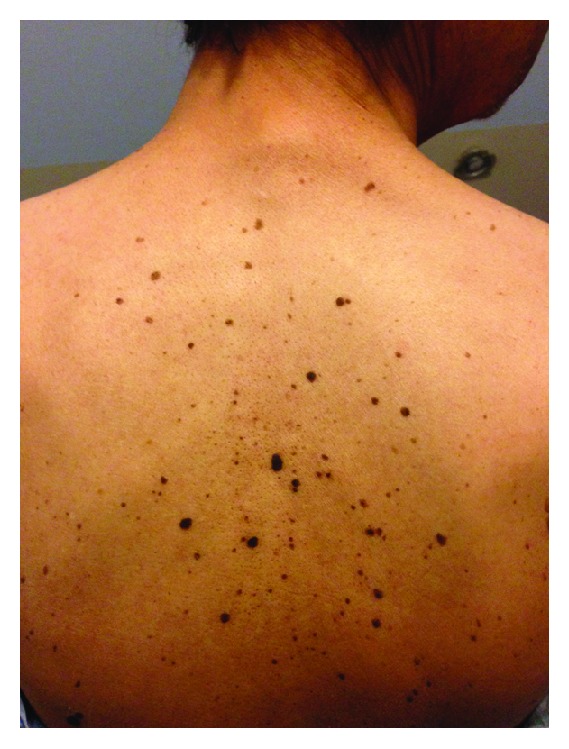
The skin lesions are seen distributed in a characteristic “raindrop” or “splash” pattern on the back.

A 65-year-old Hispanic man presented to the clinic with complaints of progressively worsening dysphagia and unintentional weight loss of 30 pounds over the preceding 2 months. Patient reported dysphagia to both solid as well as liquid food. Patient also complained of multiple worsening (both in size and in number) dark colored skin lesions on his neck and his back. His medical comorbidities included well controlled bronchial asthma and vitamin B12 deficiency. The patient admitted to chronic heavy smoking for almost 40 years. Physical examination revealed a hemodynamically stable, cachectic man (BMI: 19.4) with multiple hyperpigmented, well-demarcated and raised lesions with “stuck-on” appearance on both sides of the neck (Figure 1) and the back. The skin lesions were consistent with seborrheic keratoses distributed in a characteristic “raindrop” or “splash” pattern (Figure 2).

**Figure 3:**
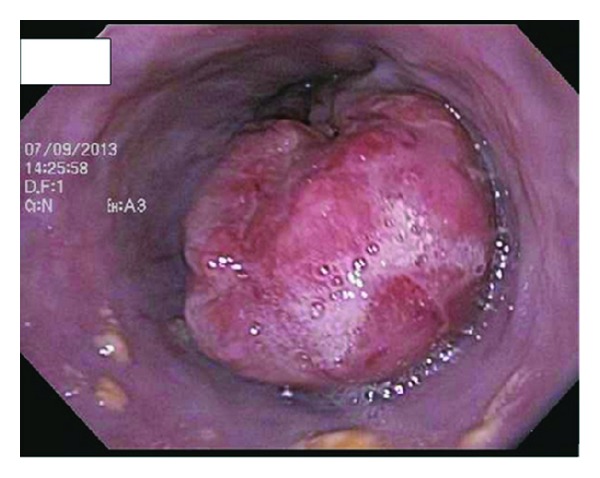
A non-circumferential, partially obstructing mass seen in the lower third of the esophagus.

**Figure 4:**
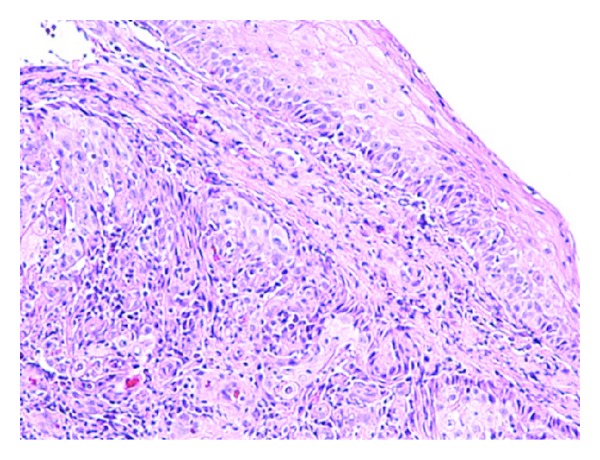
Biopsies of the esophageal mass showing poorly differentiated squamous cell carcinoma.

**Figure 5:**
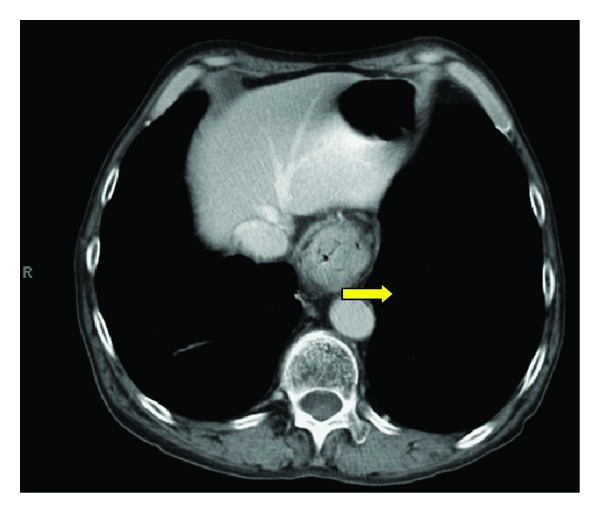
CT of chest showing a near-obstructing mass in the distal esophagus (yellow arrow).

Initial set of laboratory were remarkable only for normocytic, normochromic anemia (hemoglobin of 10.1 g/dL). He underwent a colonoscopy and esophagogastroduodenoscopy (EGD) for further evaluation of unintentional weight loss and dysphagia. Colonoscopy revealed good bowel preparation with a single 3 mm hyperplastic transverse colon polyp. EGD revealed a noncircumferential, partially obstructing mass in the lower third of the esophagus (Figure 3). Biopsies revealed poorly differentiated invasive squamous cell carcinoma (Figure 4). A subsequent staging computerized tomography (CT) scan of chest, abdomen, and pelvis revealed the mass in the distal esophagus along with several enlarged lymph nodes superior to the celiac axis (Figure 5). An endoscopic ultrasound (EUS) study revealed invasion of tumor in to the muscularis propria with perilesional lymphadenopathy.

Patient underwent surgical gastrostomy for nutritional support. Patient subsequently was started on radiation therapy and chemotherapy with weekly Paclitaxel and Carboplatin regimen. Patient underwent a repeat EGD, upon completion of 8 cycles of Paclitaxel and Carboplatin regimen that revealed significant improvement in size of the esophageal mass. However, the seborrheic keratoses did not regress in parallel.

## 3. Discussion

In spite of various arguments put forth against considering the sign of Leser-Trélat to be a true paraneoplastic phenomenon, Curth [7] provided diagnostic criteria for cutaneous paraneoplastic phenomena such as acanthosis nigricans and its frequent association-eruptive seborrheic keratoses. The pathogenesis of this rare and controversial sign is poorly understood. It has been postulated that growth factors derived from the underlying neoplasm play a role in development of these paraneoplastic eruptive disorders. The implicated growth factors include immunoreactive human growth hormone [8], transforming growth factor-*α* [9], insulin like growth factor [10], and epidermal growth factor [11]. The variance in underlying malignancy explains the different growth factors implicated in pathogenesis. It has been noted that the most common visceral malignancies associated with sign of Leser-Trélat include the adenocarcinomas of the gastrointestinal tract in about a third and lymphomas in about one fifth of patients [10]. Regression of skin lesions upon removal of underlying solid neoplasm is seen only in about half of all patients [9, 12].

Even as the sign of Leser-Trélat is most commonly associated with carcinomas of gastrointestinal tract, its association with squamous cell cancer of esophagus is exceedingly rare. To our knowledge this is only the second such association reported in the literature [13]. The most common sites of occurrence of these eruptive lesions are the trunk (in up to 76%), extremities (38%), face (21%), and neck (13%) [9]. The lesions in our patient were predominantly distributed on the back and the neck. The skin lesions of this paraneoplastic phenomenon are benign unto themselves and often regress upon treatment of underlying neoplasm. However, in our patient the skin lesions failed to regress even after significant shrinkage of tumor size upon radiation and chemotherapy.

We report this case of sign of Leser-Trélat associated with esophageal squamous cell carcinoma to highlight that an otherwise benign, asymptomatic and often ignored presentation of eruptive seborrheic keratoses could be an ominous sign that should prompt elicitation of detailed history and thorough physical examination. Any alarm symptoms or signs should be followed up by correlative testing.
